# International STakeholder NETwork (ISTNET): creating a developmental neurotoxicity (DNT) testing road map for regulatory purposes

**DOI:** 10.1007/s00204-015-1464-2

**Published:** 2015-01-25

**Authors:** Anna Bal-Price, Kevin M. Crofton, Marcel Leist, Sandra Allen, Michael Arand, Timo Buetler, Nathalie Delrue, Rex E. FitzGerald, Thomas Hartung, Tuula Heinonen, Helena Hogberg, Susanne Hougaard Bennekou, Walter Lichtensteiger, Daniela Oggier, Martin Paparella, Marta Axelstad, Aldert Piersma, Eva Rached, Benoît Schilter, Gabriele Schmuck, Luc Stoppini, Enrico Tongiorgi, Manuela Tiramani, Florianne Monnet-Tschudi, Martin F. Wilks, Timo Ylikomi, Ellen Fritsche

**Affiliations:** 1 Systems Toxicology Unit, EURL-ECVAM, Institute for Health and Consumer Protection, European Commission, Joint Research Centre, TP 580, Via Fermi 1, 21026 Ispra, VA Italy; 2 National Center for Computational Toxicology, US EPA, RTP, Washington, NC USA; 3 CAAT-Europe, University of Konstanz, Constance, Germany; 4 Regulatory Science Association, London, UK; 5 XeRR, University of Zurich, Zurich, Switzerland; 6 OECD, Paris, France; 7 SCAHT, University of Basel, Basel, Switzerland; 8 CAAT, Johns Hopkins University, Baltimore, USA; 9 FICAM, Tampere, Finland; 10 Danish EPA, Copenhagen, Denmark; 11 Green Toxicology, Zurich, Switzerland; 12 Federal Office of Public Health, Berne, Switzerland; 13 Environment Agency, Vienna, Austria; 14 National Food Institute, Technical University of Denmark, Søborg, Denmark; 15 National Institute for Public Health and the Environment (RIVM), Utrecht, Holland The Netherlands; 16 CRO, Harlan Laboratories, Itingen, Switzerland; 17 Nestlé Research Center, Lausanne, Switzerland; 18 Bayer, Leverkusen, Germany; 19 University of Applied Sciences Western Switzerland, Geneva, Switzerland; 20 Department of Life Sciences, University of Trieste, Trieste, Italy; 21 EFSA, Parma, Italy; 22 SCAHT and University of Lausanne, Lausanne, Switzerland; 23 IUF, Dusseldorf, Germany

**Keywords:** Chemical screening, Developmental neurotoxicity, Regulatory requirements, Adverse outcome pathway, Key event, Environmental hazard

## Abstract

A major problem in developmental neurotoxicity (DNT) risk assessment is the lack of toxicological hazard information for most compounds. Therefore, new approaches are being considered to provide adequate experimental data that allow regulatory decisions. This process requires a matching of regulatory needs on the one hand and the opportunities provided by new test systems and methods on the other hand. Alignment of academically and industrially driven assay development with regulatory needs in the field of DNT is a core mission of the International STakeholder NETwork (ISTNET) in DNT testing. The first meeting of ISTNET was held in Zurich on 23–24 January 2014 in order to explore the concept of adverse outcome pathway (AOP) to practical DNT testing. AOPs were considered promising tools to promote test systems development according to regulatory needs. Moreover, the AOP concept was identified as an important guiding principle to assemble predictive integrated testing strategies (ITSs) for DNT. The recommendations on a road map towards AOP-based DNT testing is considered a stepwise approach, operating initially with incomplete AOPs for compound grouping, and focussing on key events of neurodevelopment. Next steps to be considered in follow-up activities are the use of case studies to further apply the AOP concept in regulatory DNT testing, making use of AOP intersections (common key events) for economic development of screening assays, and addressing the transition from qualitative descriptions to quantitative network modelling.

## Introduction

Significant progress has been made over the past four decades in characterizing the hazards of a small number of developmental neurotoxicants. However, little effort has been made to address the challenge of assessing potential developmental neurotoxicity of thousands of chemicals currently in use (Crofton et al. 2012; Judson et al. 2009; NRC 1984, 2007). New methods, including computational modelling, hold great promise for more efficient and predictive developmental neurotoxicity (DNT) screening. In order to define a regulatory need-driven road map for an integrated testing strategy (ITS) for DNT, effective communication and discussions between various stakeholders (regulators, industry and academia) are needed. To initiate this process, the first meeting of the International STakeholder NETwork (ISTNET) was held in Zurich on 23–24 January 2014 to build consensus on the development and the use of in vitro, in silico and alternative species test methods to deliver useful data for regulatory decision-making. The meeting included 28 participants from 10 countries with a balance of experts in the regulation and management of risk (see Table 1).

**Table 1:** List of participating organizations at the First ISNET Meeting, 23–24 January 2014, Zurich, Switzerland

Bayer AG, Germany
Centre for Xenobiotic and Risk Research (XeRR), Zurich, Switzerland
Center for Alternatives to Animal Testing of Europe (CAAT-Europe), Konstanz, Germany
Center for Alternatives to Animal Testing of USA (CAAT-USA), Baltimore, Maryland, USA
Danish Environmental Protection Agency (Danish EPA), Copenhagen, Denmark
Environment Agency of Austria, Vienna, Austria
European Food Safety Authority (EFSA)
Federal Office of Public Health, Berne, Switzerland
Finish Centre for Alternative Methods (FICAM), Tampere, Finland
Green Tox, Zurich, Switzerland
Harland Laboratories, Itingen, Switzerland
Institute for Health and Consumer Protection, European Commission Joint Research Centre (EURL-ECVAM)
IUF—Leibniz Research Institute for Environmental Medicine, Dusseldorf, Germany
National Food Institute, Technical University of Denmark (DTU), Søborg, Denmark
Nestle AG, Switzerland
Organisation for Economic Co-operation and Development (OECD), Paris, France
Regulatory Science Association, UK
National Institute for Public Health and the Environment (RIVM), Utrecht, Netherlands
Swiss Centre for Applied Human Toxicology (SCAHT), Basel, Switzerland
University of Applied Sciences Western Switzerland, Geneva
University of Trieste, Department of Life Sciences, Trieste, Italy,
University of Lausanne, Lausanne, Switzerland
National Center for Computational Toxicology, US Environmental Protection Agency (US EPA), NC, USA

During the meeting, a review of animal-based test methods currently used for developmental and adult neurotoxicity evaluation for regulatory purposes led to the conclusion that these methods are not being routinely used due to high costs and the use of large numbers of animals. A new testing paradigm is needed that can overcome these limitations.

In vitro/in silico modelling approaches are needed in order to provide value-added data for regulatory purposes, including:

- Reduction in animal numbers and animal suffering for testing,
- Reduction in testing costs,
- Increased testing by using high-throughput systems (HTSs) in order to estimate environmental hazards to human health from thousands of substances and mixtures on the market in a reasonable time frame,
- Improve testing of environmentally relevant mixtures to discriminate synergistic or antagonistic effects,
- Improve practical bio-monitoring of environmental media to detect effects of unknown contaminants that would not appear with analytical chemical measurements,
- Improve testing of the multiple physicochemical variants and size distributions of nanomaterials,
- Cost-effective testing of low production volume chemicals, metabolites, degradation products, impurities and others, for which no legal data requirements can be established for practical reasons.

The main focus of the meeting was to discuss how regulatory requirements for DNT testing might be met by alternative approaches such as in vitro test methods, quantitative structure–activity relationships (QSARs), read across, and application of the new adverse outcome pathway (AOP) concept. This paper describes the main focus of the first ISTNET meeting: how to increase the use of alternative sources of data in DNT risk assessment and risk management decisions.

## Current regulatory in vivo procedures

During the development of chemicals to which humans may be exposed, mainly drugs and pesticide, a wide range of hazard studies are performed. These studies, combined with exposure information, allow characterization of margins of safety/exposure for humans. Regulatory studies are designed to allow us to complete an evaluation as possible of any pharmacological or toxicological effects which may impact human health. The majority of these studies are performed in laboratory animals and comply with international regulatory guidelines. The ultimate objective of this testing is to enable safer use of chemicals, through hazard identification and risk assessment.

Regulatory guidelines for studies that generate information about developmental neurotoxicity have been issued by OECD and many national regulatory agencies (e.g. US EPA; Japan Ministry of Agriculture, Fisheries and Food [JMAFF]). The three primary OECD guidelines covering life stage-dependent neurotoxicity are OECD 424—neurotoxicity study in rodents (OECD 1997), OECD 426—developmental neurotoxicity study (OECD 2007) and OECD 443—extended one-generation reproductive toxicity study (OECD 2011). There is also an accompanying guidance document (OECD Guidance Document for Neurotoxicity Testing (OECD 2004) on study design and selection of additional or alternative in vivo or in vitro test methods. The purpose of the OECD guidelines is to identify chemicals that permanently or reversibly affect the nervous system, to characterize any chemical-induced alterations in the nervous system and to estimate dose levels (points of departure) for regulatory uses. The studies mainly utilize rodents, with the rat being the preferred species, although other species may be used with justification. Specific endpoints to evaluate functional, behavioural and morphological effects of the nervous system in all study types include:

1. Detailed clinical observations in the home cage and open field,
2. Neurofunctional tests including motor activity,
3. Neuropathology using perfusion-fixed tissues.

Additional testing specifically for offspring that have been exposed to utero and early lactation includes sensory function testing, sexual maturation (OECD 426 and OECD 443), assessments of behavioural ontogeny and learning and memory (OECD 426).

The functional tests and clinical observations in these guidelines are similar to those specified in OECD Guidelines 407 (OECD 2008) and 408 (OECD 1998) (rodent 28- and 90-day repeated dose oral toxicity studies), but employ a larger sample size than advised by the OECD Guideline 407, calling for more frequent evaluation of functional tests, and require that observations are conducted without the knowledge of treatment.

Typically, specific neurotoxicity studies are not required if there are no indications of neurotoxicity from standard regulatory repeat-dose toxicity tests or human data (e.g. EU chemical and pesticide regulations 1907/2006 and 283/2013), but there are some national differences (e.g. the US EPA requires adult neurotoxicity studies for all pesticides). Assessment of developmental neurotoxicity in OECD 443 or 426 studies is usually requested when data from standard adult and/or reproductive toxicity studies indicate a possible concern for neurotoxicity. When guideline neurotoxicity studies are conducted, more detailed tests of nervous system function are possible (as described in OECD Guidance Document for Neurotoxicity Testing (2004), but are seldom conducted in practice. For example, guidance on the US EPA Neurotoxicity Screening Battery for Pesticides and Chemicals (OPPTS 870.6200 and 40 CFR 799.9620, respectively), which is closely aligned to OECD 424, notes that “*there is no clear consensus concerning the use of specific behavioural tests to assess chemical*-*induced sensory*, *motor*, *or cognitive dysfunction in animal models*”.

The comparison of data for known human neurotoxicants indicates that experimental animal data, such as generated in regulatory neurotoxicity studies, are frequently predictive of a neurotoxic effect in humans (Chang and Dyer 1995; Rees et al. 1989, 1990; Schaumburg and Spencer 2000). Although most clinical neurotoxicity signs can be reproduced in animal models using rodents, this is not always the case. For example, in a 90-day rat neurotoxicity study (OPPTS 870.6200, equivalent to OECD 424), ethylbenzene produced inconsistent changes in auditory startle which were considered not treatment related (Li et al. 2010). However, several non-regulatory studies have reported hearing loss in rats (but not guinea pigs), with irreversible loss of auditory function and associated loss of cochlear outer hair cells; therefore, the current EU proposed labelling is Specific Target Organ Toxicity Repeated Exposure (STOT RE 2); H373 “*Warning: May cause damage to hearing organs through prolonged or repeated exposure*” (ECHA 2012).

A detailed review of the performance of data submitted to the EPA using the DNT guideline (Makris et al. 2009) concluded that the current guideline “*represents the best available science for assessing the potential DNT in human risk assessment, and data generated by DNT studies are relevant and reliable for this assessment*”. As the studies include parameters that are not assessed in other guideline studies, the authors concluded that these guidelines are capable of detecting changes in the nervous system not found with other guidelines (e.g. sub-chronic, reproductive toxicity). However, as previously mentioned, these guidelines are very resource intensive in terms of animals, time and overall cost (Rovida and Hartung 2009; Tsuji and Crofton 2012) and have been used only for a very limited number of pesticides and industrial chemicals. This highlights the pressing need for alternative methodologies that can more rapidly and cost-effectively screen large numbers of chemicals for their potential to cause DNT or investigate mechanisms to provide information on human relevance (Crofton et al. 2012). Such information can be used to help prioritize compounds and/guide the design of further, possibly less resource-consuming in vivo tests.

## Regulatory perspectives on current DNT testing

Regulatory authorities face challenges with regard to whether potentially hazardous substances are sufficiently tested for adverse effects on the nervous system, in particular the developing nervous system. For example, a recent comprehensive review of Grandjean and Landrigan (2014) raised concern about the increased frequency of neurodevelopmental disabilities such as autism, attention deficit hyperactivity disorder and dyslexia among children could be caused by exposure to industrial chemicals, which have not been sufficiently tested for DNT. Other evidence comes from epidemiological studies that associate exposure to some pesticides and effects on children’s neurological development (Horton et al. 2011; Rauh et al. 2011, 2012). Although a more systematic review (Burns et al. 2013) failed to find strong evidence for such associations. It is important to note that regulatory DNT guideline studies have only been required for some pesticides, and pesticides are only a small portion of the total universe of untested chemicals, some of which are known or suspected developmental neurotoxicants (Grandjean and Landrigan 2006).

**Fig. 1:**
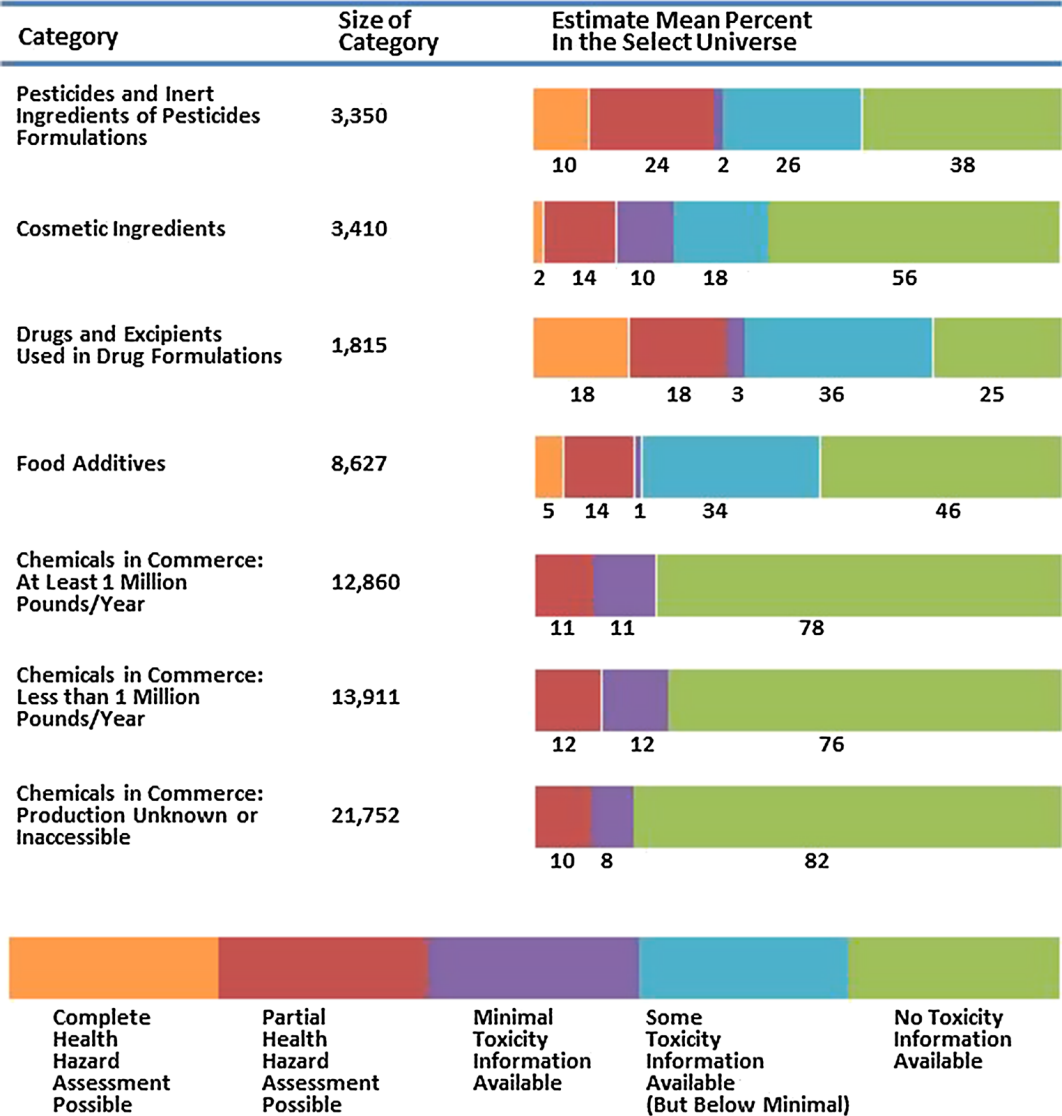
Summary of data available for conducting health-hazard assessments of chemicals (adapted and modified from NRC 1984; reprinted from Crofton et al. 2012)

A major challenge for regulatory authorities is the lack of adequate DNT data for the thousands of chemicals in commerce. Historically, the problem of lack of data was recognized as far back as 1984 when the US National Academy of Science (NRC 1984) released a report on the status of toxicity testing and estimated that there could be as many as 64,000 chemicals in commerce and that the majority either lacked, or had inadequate test data, to estimate hazard potential (Fig. 1). More recently, Judson et al. (Judson et al. 2009) estimated that there were approximately 20,000 high-priority chemicals based on known bioactivity (e.g. pesticides), high production volumes or widespread exposure potential (e.g. drinking water contaminants). Considering that only 100 or so chemicals have actually been tested using regulatory DNT guidelines (Makris et al. 2009), the challenge facing the field of DNT testing is daunting. An alternative to currently available regulatory test methods that reduces both time and costs while maintaining or improving our understanding of DNT potential is urgently needed.

## Regulatory needs for DNT data

In the context of the EU regulation of chemicals (Registration, Evaluation, Authorisation and Restriction of Chemicals (REACH), currently the main indications for neurotoxicity testing refer to clinical signs, functional observational test battery and neuropathology in standard repeated-dose studies. Additional information on neurotoxicity and/or DNT may be required if cause for concerns appears from these standard repeated-dose or reproductive toxicity studies (developmental or 1- as well as 2-generation studies) or related to a specific modes of action or chemical structure information (EU Regulation 528/2012, Annex II, 18.13.2. (EU 2012); EU Regulation 283/2013, Annex 5.6.2 (EU 2013a). The legally limited regulatory requirements are likely due, among others, to the high costs, large animal numbers and the scientific dispute on the uncertainty of standard DNT studies (OECD 2008; Smirnova et al. 2014).

This regulatory climate contrasts with the potentially strict downstream consequences. Though it is not explicit in the legal text of the EU Classification, Labelling and Packaging (CLP) regulation, clear evidence of DNT, at least theoretically, could trigger classification as reproductive (developmental) toxicity category 1A or 1B. This classification could result in a status as a substance of very high concern with authorization restriction consequences. In the context of the Biocides and the Plant Protection Regulation such substances would be candidates for substitution (if the substance has not been excluded according to the criteria laid down in point 3.6.3 for PPP, Art. 4(1), BPR Art 5(1) c). Alternatively, a DNT concern could theoretically result in classification for STOT RE with (usually more limited) legal downstream consequences. Importantly, the Biocides Regulation explicitly mentions that products with DNT concern shall not be marketed to the general public (BPR Art 19(4)e). Thus, while the attention currently paid by regulators to potential DNT effects is somewhat ambivalent, with limited upfront data requirements, it can have important regulatory consequences.

In vitro methods currently provide great promise as more cost-effective methods for rapid screening of chemicals for DNT potential (Bal-Price et al. 2012; Crofton et al. 2011). In addition, these methods are likely to provide the data required to prioritize the vast numbers of untested chemicals for further in vivo testing. The use of in vitro and in silico tests for DNT to trigger standard DNT animal tests without consideration of their intrinsic value is clearly indefensible. However, it should be acknowledged that in vitro/in silico modelling data are not yet sufficient to satisfy all regulatory needs. Therefore, in the short-term regulators need validated batteries of in vitro/in silico methods that, as far as necessary, can be used to prioritize chemicals and for targeted in vivo follow-up studies. A common understanding of the uncertainties associated with new in vitro and in silico modelling approaches in comparison with in vivo data should be developed to support validation of new in vitro/in silico approaches and build confidence in broader regulatory use of in vitro/in silico modelling data. Finally, a decision framework is needed on how the in vitro/in silico data will be used and for what regulatory purposes (e.g. screening with the aim of further testing, category formation and read across of standard animal data) or regulation in terms of classification or risk assessment (Patlewicz et al. 2014).

As an initial step towards this goal, we propose the use of an integrated testing and evaluation platform in which regulatory problem formulation drives testing (Crofton et al. 2012) allowing more efficient use of resources, targeting data generation directly to regulatory need. Suggested criteria for an approach are listed below:

- A battery of in vitro test methods and in silico models is needed that cover all relevant key neurodevelopmental processes.
- In vitro testing systems should have some metabolic capacity relevant for humans.
- Data and models should be developed that increase regulatory confidence in extrapolation from in vitro to in vivo.
- The various regulatory uses of DNT in vitro data and in silico models must be considered when developing a “fit for purpose” validation framework
- QSARs predicting molecular initiating events for which highly reproducible in vitro test data are available may be more relevant than QSARs predicting apical animal study effects with less clear reliability and relevance.
- Computation DNT models are needed which discriminate in vitro effects that lead to adverse downstream outcomes from those for which cellular- and tissue-level compensatory processes preclude adverse outcomes. This is a key regulatory need for the establishment of human reference doses for risk assessments. To ensure that regulatory actions are based on the best available science, constant vigilance is needed over the rapid advances in neuroscience and computational modelling that can be incorporated into improved in in vitro *and* in silico DNT methods.

## Introduction of the AOP concept

The adverse outcome pathway (AOP) concept provides a framework for representing existing knowledge concerning the linkage between the molecular initiating event (MIE) and an adverse outcome at the individual or population levels (Ankley et al. 2010; OECD 2013). This framework relies on understanding correlative and causal relationships between the MIE, in which a chemical interacts with a biological target, resulting in a sequential series of measurable key events (KEs), which are cellular, anatomical and/or functional changes in biological processes that ultimately result in adverse outcomes manifesting in an individual organisms and/or a population. By definition, AOPs span multiple levels of biological organization that are often depicted as linear processes. However, biological systems involve complex interactions between multiple processes, and thus are in reality not linear. Development of AOPs vary in the level of detail and linearity characterizing the pathways and AOPs can vary substantially, both as a function of existing knowledge and risk assessment needs. Watanabe et al. (2011) provides an example of the development of an AOP for over activation of the kainate receptor leading to neuronal cell death and impairments in cognitive function. Earlier examples of indirect effects on the developing nervous system include the use of the mode-of-action (MOA) framework pathway analysis for developmental neurotoxicity that results from disruption of thyroid hormones during foetal and early post-natal life (Crofton and Zoeller 2005). Ideally, causality across AOPs is approached not only in a qualitative, but also in a quantitative way relating exposure to the adverse outcome (OECD 2013; Vinken 2013; Meek et al. 2014).

The limited number of DNT AOPs has hampered both judgement of the predictive ability and regulatory use of high-throughput in vitro DNT data. To address this gap, a EURL ECVAM-SEURAT-1 workshop was held in March 2013 in Ispra (Italy), which applied the AOP framework to adverse health outcomes associated with life stage-specific neurotoxicity. The output of the workshop was the identification of ten putative AOPs (Bal-Price et al. 2015) for both neurotoxic and developmental neurotoxic outcomes. While these AOPs are not yet fully described, they do function to stimulate more detailed AOP development via identification of data gaps and discrimination of correlative verses causative relationships between KEs. This workshop report also highlighted that the importance of the AOP concept in guiding development of in vitro methods, and the use of resulting data streams cannot be overstated.

## Perspectives on how AOP concepts inform the use of in vitro methods

### Use of the AOP framework for chemical category formation

In order to understand the strength of the AOP concept in DNT testing, it is important to consider the tools available (or under development) that will use in vitro/in silico information. One application of in vitro methods will be to support chemical category formation (Roberts and Patlewicz 2014) not only with regard to toxicity categories but also more broadly such as the grouping of chemicals with similar structures and biological activities. This information could then be used for regulatory read-across activities (Patlewicz et al. 2014). The AOP concept can be an important tool that facilitates generation of the data needed for the formation of chemical categories: chemicals can be grouped according to their MIEs, and sometimes common KEs. AOPs provide a strong biological/pathophysiological rationale to compound classification, which is usually based on chemical structures correlated to apical endpoints from animal experiments. AOP-based chemical category formation has the potential to add a value for DNT testing due to the complex nature of the underlying biology that is currently inadequately captured by chemical category formation (structure or reactivity).

### Use of AOP for the incorporation of in vitro DNT data into integrated testing strategies

**Fig. 2:**
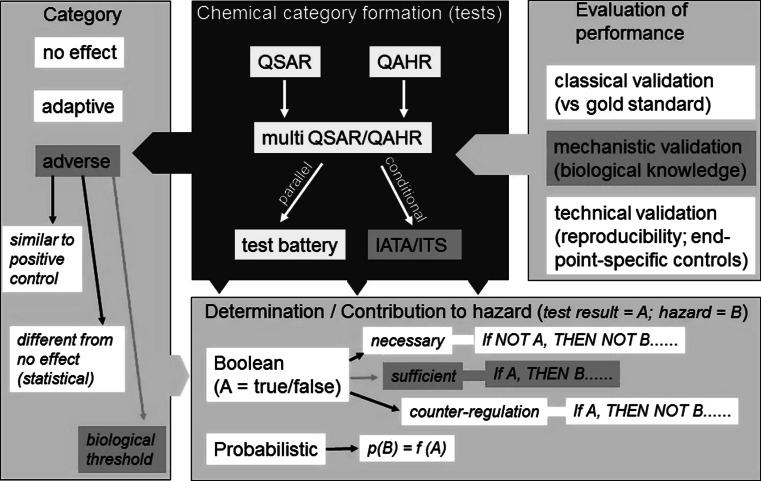
Chemical category formation and toxicant assessment. A traditional chemistry-driven approach of classification/category formation is based on quantitative structure–activity relationships (QSAR). A complementary approach uses the actual activity of a compound (i.e. the effect in a test system) to relate it to its potential hazard (QAHR). Multiple QSAR/QAHR may be combined into test batteries or into integrated testing strategies (ITS)/integrated approaches to testing and assessment (IATA). All category formation approaches require some form of evaluation of their performance. This may take the form of a classical validation or mechanistic validation or merely a technical validation. Simple classification outcomes are “no effect”, “adverse effect” or “adaptive effect”. An adverse effect may be defined in different ways (*left bottom*). At the *bottom right*, different logical approaches to hazard prediction within the context of a biological pathway or AOP are indicated. In probabilistic risk assessment, the likelihood of a certain hazard (p(B)) would be a function of the test outcome (f(A)). The *orange boxes* exemplify a specific choice of approaches that may be used in the context of test structuring according to the AOP concept: one may choose to take the approach of an ITS that is mechanistically validated. Hazard would be defined on the basis of the biological thresholds relevant to the key events of the AOP. Focus for hazard prediction would be on events that are sufficient by themselves to explain/result in hazard (color figure online)

The concepts that underlie the AOP framework can guide more effective inclusion of in vitro test data into integrated testing strategies (ITSs). For example, read-across and toxicity classification models can be vastly improved when large amounts of in vitro data are available from high-throughput testing. Until now, these models have been mainly based on limited animal data available for some members of a read-across group or on chemico-physical properties and structural chemical similarities (e.g. Cronin 1996; Estrada et al. 2001). Much richer data sets obtained from in vitro bioactivity testing allows for empirically based correlations between chemical structure and hazard in quantitative activity-hazard relationships (QAHRs) (OECD 2007), also sometimes referred to as quantitative activity–activity relationships (QAARs). Combinations of the quantitative structure-based models into ITSs (Hartung et al. 2013b), sometimes (OECD 2012a, b) also described as Integrated Approaches to Testing and Assessment (IATA) (Fig. 2), are technically demanding, but an important goal, since it may provide a more objective and robust approach compared to ad hoc categories and groups formed based on a narrative of available data. The AOP concept can assist in the selection of the most important tests to use in IATA, reflecting more appropriate coverage of MIEs and KEs (Tollefsen et al. 2014). Such a testing strategy for a neurodevelopmental process will have to reflect different stages of development as well as early and late processes conferring to brain formation. Which and how many of the currently available in vitro test systems are necessary to cover the most important neurodevelopmental KEs in ITS has to be revealed by analyses of chemical testing results in the future.

### Guidance for new approaches to validation of DNT assays

All new methods for in vitro screening, including DNT, require some form of evaluation of their performance. Demonstrating that a DNT test method or model provides scientifically valid information is critical for regulatory acceptance and use. Traditionally, validation of methods is based on the evaluation of the reliability, i.e. reproducibility of the method (within and between laboratories) and the relevance of the method, i.e. how well it predicts the “true” result (Hartung et al. 2004; Leist et al. 2010, 2012b; OECD 2005, 2013). However, it is increasingly recognized that for some complex endpoints, it is difficult to define “true” results, especially when the goal is the prediction of adverse outcomes in humans. Empirical data on the impact of environmental chemicals on health effect are very limited and usually come from retrospective rather than prospective epidemiological studies. Since the aim is to scientifically improve human hazard assessment, it is not the animal test results that should be predicted, but human toxicology (Paparella et al. 2012). Moreover, while data from animal testing are more readily available, there remain a number of well-recognized uncertainties in extrapolation to humans. The answer to the question of how to define “true” results will probably come from the concept of “mechanistic validation” (Balls et al. 1995; Hartung et al. 2013a; Leist et al. 2012a) that in essence requires defining the biological relevance of the pathway of toxicity (Hartung and McBride 2011; Kleensang et al. 2014) using a few substances which are well characterized in humans (Kadereit et al. 2012). AOPs can inform this process using new DNT methods by mapping out the most important biological pathways and linking them to adverse outcomes. Of course, within- and between-laboratory reproducibility estimates are still important for validation, and hazard data should be further integrated with distribution estimates of cellular concentrations on the basis of physiologically based toxicokinetic models (Blaauboer et al. 2012; Judson et al. 2011; Leist et al. 2012b). Mechanistic validation, based on the biological knowledge and different precise interventions, would be based not only on the correlation of results of test and reference model, but on the mechanism underlying the AOP of DNT hazard manifestation (Hartung and McBride 2011; Leist et al. 2012a).

As already described by the European Validation of Alternative Methods (ECVAM) in 1995 (Balls et al. 1995) and taken up in OECD GD34, validation of any method should be carried out towards a specific purpose. The purposes can be multifold and are not solely related to full replacement of the regulatory animal test. As the emphasis in recent times is the development of IATA which address potential adverse health effects in humans, without extrapolating from animal data, then the purpose of validating in vitro or in silico methods is typically to assess the value and associated uncertainty of information derived from the method in the context of an IATA. Moreover, if the IATA is based on mechanistic rationale, derived, for example, from knowledge described as an AOP, then the value of the method will be related to its (mechanistic) relevance to key events and the reliability (reproducibility) of the data it generates.

For in vitro DNT methods, validation requirement (Hartung et al. 2004) may be relaxed when the goal of testing is screening of chemical library to identify substances of highest concern for further testing. This approach has been termed screening for prioritization. Such screening exercises can be accomplished with high-throughput screening (HTS) methods. In such cases, a technical validation may be appropriate to establish reproducibility of the method and to verify its expected behaviour when exposed to endpoint-specific control compounds (e.g. Crofton et al. 2012). The latest proposal for this field was published by a group of experts with regard to HTS validation (Judson et al. 2013). This group acknowledged the need for different validation processes that are “fit for purpose”, meaning that the degree and type of validation will vary depending on the regulatory purposes for which data may be used. In the end, the ability of in vitro test results to predict the effect of exposure on human physiology must always be demonstrated. However, as human DNT data for environmental chemicals are sparse and testing in humans is not possible, one approach towards scientific validation is the comparison of human in vitro data with analogous rodent in vitro assays, which can then be directly compared to rodent in vivo data and extrapolated to the in vivo human (Kienhuis et al. 2009).

### Interpretation and combination of test results

AOPs hold great potential to impact the manner in which in vitro DNT data can be interpreted. As stated earlier, AOP provides correlative or causative links between MIEs, KEs and adverse outcomes (Landesmann et al. 2013; Ankley et al. 2010; OECD 2013). This linkage, based on empirical data and biologically relevant knowledge, provides more certainty for regulatory use of in vitro DNT data. In addition, information from multiple tests for a pathway that are consistent, will, enhance the hazard assessment. In addition, AOPs provide a framework for the discrimination of in vitro changes that are adverse (e.g. toxicologically relevant and predictive of the adverse outcome) from those that are adaptive (e.g. related to compensatory processes that do not lead to an adverse outcome (Boekelheide and Andersen 2010). Three fundamental and practically applicable approaches are available to define adverse effects. A chemical may be classified as adverse (1) when its effect is beyond the noise level of inactive compounds, (2) when its effect is similar to that of a positive control compound or falls in the range of positive controls, or (3) when its effect is beyond a meaningful, biologically defined threshold. Combinations of the approaches are possible (Fig. 2).

In this context, the need to differentiate adaptive from adverse effects in vitro requires further discussion. The distinction may not be as important as actually perceived, especially for screening of chemicals for prioritization. While effect definitions that do not account for potential compensatory reactions will probably lead to more conservative toxicity estimates at the screening level, this is in agreement with the precautionary principle that the burden of proof that it is not harmful falls on those taking action. This may also be recognized in risk assessment by using different uncertainty factors. In order to decide on which approaches to rely on for regulatory decisions, it is essential to characterize the nature of effects found in standard animal testing in comparison with the uncertainty of the new in vitro *and* in silico modelling approaches. The ultimate goal is to be able to characterize in vitro and in silico DNT information for the use in regulatory decision and to do so with a focus on the potential of the data to accurately predict adverse consequences.

### Transition from apical endpoints (downstream consequences) to early (upstream, triggering) events related to toxicity

**Fig. 3:**
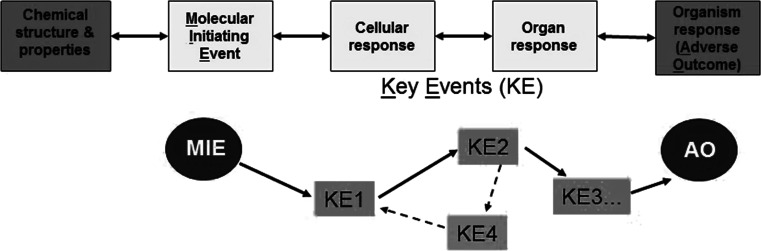
Concept of adverse outcome pathways (AOPs). A complete AOP spans the events linking a chemical’s structure and properties to the adverse outcome (AO) it triggers in an organism. The decisive first step is a defined molecular initiating event (MIE), an interaction of the chemical with a target. This triggers cellular responses through metabolic and signalling pathway perturbations; these cellular responses result in changes in tissues, organs and the organism. A pivotal element of the concept is the assumption of key events (KE). Complexity may arise, when reality suggests that one KE is directly upstream of two or more other KE, or when one of the KE is involved in a feedback loop

The transition of animal-based hazard assessment to non-animal methods requires a change in the concept of toxicity testing (NRC 2007; Hartung 2009; Hartung et al. 2012; Leist et al. 2008, 2012a, b; Smirnova et al. 2014). Animal testing is based on parallel assessment of apical endpoints (Blaauboer et al. 2012), adverse outcome measures that most often represent relatively late events in toxicological damaging cascades. In quality assurance (QA) of industrial production of, for example, drugs or vaccines, this would correspond to the principle of end product control. The AOP concept is radically different (Fig. 3), and in an industrial QA, it would correspond to the more modern principle of process control, i.e. assessment of each individual step at which a potential quality reduction may occur. These steps are termed key events (KEs) in the AOP concept, and it is assumed that a series of KEs link the molecular initiating event (MIE) to the final adverse outcome (AO). KE may be triggered by various cellular responses such as alterations in signalling or metabolic pathways. Triggering of a KE is assumed to be necessary for the next step to occur and for toxicity to manifest itself. Activation of the full cascade of KEs is considered to be sufficient for triggering the AO. The use of the AOP concept for risk assessment implies the setting up of assays for MIEs or KEs of DNT pathways, to provide a consistent rationale that a given test chemical does or does not affect the AOP. In theory, this provides a scientifically sound and potentially more robust and sensitive approach to toxicity testing than the use of apical endpoints in animals. However, there is still work required concerning the quantitative use of the concept. The situation is relatively straightforward if the extent to which a MIE or KE is altered and is known to be sufficient to trigger the final AO (Fig. 3). It is assumed that the AO occurs only after a biologically meaningful overall threshold has been passed (Boekelheide and Andersen 2010; Ramirez et al. 2013). However, the situation is more complex, when the MIE (or KE) is necessary, but not sufficient to generate and AO, or when feedback loops or compensatory processes exist between KEs. Recently, web-based applications like the OECD sponsored AOP-Knowledgebase (https://aopkb.org/), the AOP-Wiki (http://www.aopwiki.org), Effectopedia (http://www.effectopedia.org) and Human Toxome knowledgebase (http://www.humantoxome.com) have been developed to facilitate AOP development.

**Fig. 4:**
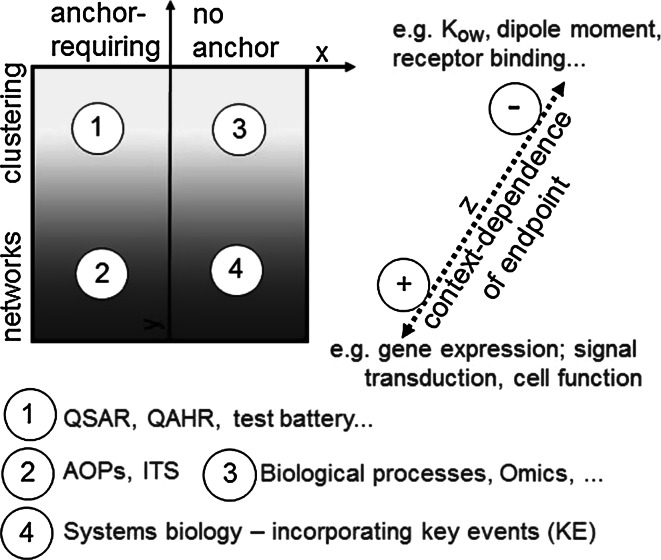
Anchor and context dependence of different chemical assessment methods. Different approaches for hazard testing and classification may be distinguished by their dependence on anchoring (*x* *axis*), i.e. relating the results to other information not delivered by the test method. For instance, most classical assays and models (QAHR/QSAR) require high numbers of already known compounds for calibrations. In contrast to this, testing of biological processes (e.g. neurite growth) does not necessitate such information. Approaches may also be distinguished (*y* *axis*) by the extent to which they use networks or simple clustering approaches to categorize information form multiple sources. A third dimension (*z* *axis*) distinguishes methods by the context dependence of the endpoint measured. For instance, receptor binding constants or the polarity of a compound would be only to a small extent dependent on the assay used. In contrast to this, gene expression changes triggered by a compound will depend on the cell type, the culture conditions and many other factors

For descriptions of full DNT AOPs, anchoring information is required: both the MIE and the AO need to be known (Bal-Price et al. 2015; OECD 2013). This may sound extremely trivial, but given the situation of DNT in humans, very little quantitative data are available on AO with the relevance to DNT, and for most neurological disorders (e.g. autism, ADHD, Alzheimer’s), the MIE is unknown. Moreover, it is often very difficult to relate the AO in humans to defined organ changes measurable in experimental systems, the so-called toxicity endophenotypes (Balmer and Leist 2014; Kadereit et al. 2012; Smirnova et al. 2014). Therefore, it is important to consider, which approaches may also work in an anchor-independent way (Fig. 4), and how partial AOPs may be applied in DNT testing.

### Developmental processes underlying the MIEs and KEs of DNT AOPs

The lack of knowledge of the MIEs for many DNT AOPs leads to the question whether one could work on the middle part of an AOP only and still obtain meaningful information. It appears that such approaches are indeed already available: for instance, there are tests for key biological processes of neurodevelopment that can be measured using a variety of biochemical or neuro-functional read-outs. The key processes in DNT include neural stem/progenitor cell proliferation, apoptosis, cell migration, cell differentiation into neurons and glia cells, neurite outgrowth, myelination, dendrite and synapse formation, and the endpoints use either cell biological and biochemical endpoints or appropriate transcriptomics/epigenetics/metabolomics methods (Balmer and Leist 2014; Baumann et al. 2014; Gassmann et al. 2010a; Harrill et al. 2011; Hayess et al. 2013; Kuegler et al. 2009; Moors et al. 2007, 2009; Ramirez et al. 2013; Hogberg et al. 2013; Pallocca et al. 2013; Theunissen et al. 2012a; van Thriel et al. 2012; Wayman et al. 2012). Other available approaches include neuronal cultures using defined conditions which ensure reproducible maturation stages of neuronal development in vitro, similar to those observed in vivo (Baj et al. 2014; Cáceres et al. 2012). These developmental stages are defined by morphological endpoints, such as differentiation of the axon, establishment of the apical dendrite, but also functional endpoints such as onset of spontaneous activity (Baj et al. 2014; Hogberg et al. 2010) or conversion of GABAergic-mediated neurotransmission from excitatory to inhibitory (Ben-Ari and Spitzer 2010). High-throughput analysis of morphological or functional endpoints is feasible by high-content microscopy or automated electrophysiology testing, respectively. Importantly, it should be noted that while many automated morphological or functional analytic systems have been applied to 2D neuronal cultures, the analysis of 3D cultures presents additional challenges that need still to be addressed (Alépée et al. 2014). The key assumption for this approach is that the KEs tested by these methods are considered to be predictive of adverse effects (Fig. 3). For example, a chemical that blocks synapse formation will lead to adverse developmental neurological outcomes. However, most importantly, this conclusion is valid, even though the exact AO and the MIE are unknown (see “Interpretation and combination of test results” section for a discussion concerning compensatory processes and adverse outcomes).

An additional goal in the development of DNT AOPs is the quantitative incorporation of developmental events or cellular determinants of key biological processes in a systems biology model of the cell (Hartung et al. 2012). This would ideally be built from knowledge of the underlying biology and the pathway maps (see Fig. 6 in Kleinstreuer et al. 2013). The information requirements and bio-informatic resources needed for such an approach may be large, but it has been discussed that the use of such models may then allow better identification of developmental events and the design of follow-up studies that are target or tailored for specific purposes (Rossini and Hartung 2012). Such a tailored approach would then be useful for routine testing. Concerning this issue, it will need to be determined, whether the data from tailored assays are highly context dependent, i.e. whether complex assay systems are required to model the in vivo biological context to a large extent, or whether more simple assays of standard cell biological changes and biochemical reactions would be sufficient. With all these considerations, it needs to be remembered that the strategy for in vitro-based and AOP-oriented test strategies will require recursive steps. For instance, an immediate start may be provided by the approaches that are relatively anchor independent (Fig. 4). With time, their use would provide better anchors (availability of established tool compounds; knowledge on AO on the tissue level) to allow better use of anchor-dependent approaches. In the same vein, the comparison of highly context-dependent assays with biochemically oriented test batteries may help to identify more simplified cell-based assays and/or a more relevant set of biochemical endpoints for screening purposes. A second consequence of these considerations is that the developmental “stage dependence” is a very important concept of DNT AOPs and needs to be considered for the selection of positive and negative control substances for test systems.

### Context dependence of DNT assays

DNT AOPs could be used to “bundle” sets of tests to better understand how integrated systems response to multiple chemical stressors. Therefore, it is important to determine how data from test methods for KE within an AOP, as well as between AOPs, interact or not. In vitro approaches may be categorized according to the extent to which they inform networks or simple clustering approaches to categorize information from multiple sources. For instance, QSAR/QAHR (Quantitative Activity-Hazard Relationships) approaches are at present used in parallel and the tests require individual validation and respective control compounds (Fig. 2). It is not yet clear how situations should be handled when the results of a test battery suggest that certain MIEs or KEs of an AOP are triggered with high potency, but others with low potency. Practice and experience is needed to determine whether the last KE is the most relevant, or whether other decision rules should be applied. The situation is different for a fully integrated systems biology approach. The entire prediction process would be network based, and the performance evaluation could only be done for the entire set-up (Fig. 4). Notably, also within an ITS, individual tests might have a fundamentally different context dependence. We still do not know whether an ITS based on “biochemical/biophysical-only” assays would work for DNT, and different toxicological fields may have different requirements. For instance, acute cytotoxicity may be deduced from test systems that are relatively context independent (protein denaturation, mitochondrial inhibition or membrane permeabilization), while mapping of an AOP for DNT would require tests that are extremely context dependent (e.g. working in a cerebellar neurons, but not in cortical neurons or glial cells). For the practical use of the AOP concept and its further development, experience will show how assays of different complexity and context dependence can be combined, and whether this will be in fully integrated networks or in clusters of parallel tests.

### Applying DNT AOP to chemical mixtures

Mixtures are still one of the greatest unsolved conceptual problems in toxicology. The prediction of their toxicity has been mainly based on apical endpoints for the individual compounds. However, the AOPs for various components in relevant heterogeneous environmental media superimposed on each other. This leads to considerable uncertainties if the mixture toxicity is predicted just on chemical analytical data and individual substance toxicology. Two alternative approaches are offered by the use of in vitro test systems within the AOP concept. The first uses the lower cost and higher throughput of some test systems to actually test the hazard of mixtures themselves, instead of the individual components only (Cavallin et al. 2014). This is often not possible with animal experimentation for reasons of cost and logistics. The second approach puts the a priori prediction of mixture effects on a more solid scientific basis. Research into the prediction of mixture effects of potential DNT toxicants from KE of AOP might lead to a large advance in safety sciences, where predictions on mixture effects are of high importance.

## How in vitro DNT tests can be used in regulatory decision-making

Because of the chemical potential to produce adverse health effects such as DNT, chemical hazards relevant for human safety need to be identified and managed. From a regulatory perspective, this includes the approval of new chemicals for pharmaceutical, food, agricultural and/or industrial applications, establishing limits for chemical contaminants in water, air and food, handling of incidents/crisis triggered by incidental chemical contaminations.

It is acknowledged that risk assessment plays an important role in chemical management and regulatory decision-making. Risk assessment is chemical specific. It compares exposure estimate with a health-based guidance value, which could be either a safe level or, if not available, an exposure level associated with a predetermined level of risk (WHO 1999).

Traditionally, the establishment of a safe level of exposure (called hazard characterization) has been based on toxicological studies conducted according to internationally recognized regulatory guidelines addressing an array of endpoints (e.g. subchronic/chronic toxicity, reproductive toxicity, teratogenicity and carcinogenicity). It is important to note that DNT studies are rarely available in classical regulatory toxicological databases, therefore raising the question of the adequacy of currently used health-based guidance values to cover this specific endpoint. As discussed above ("Regulatory perspectives on current DNT testing" section), new in vitro DNT methods (including in vitro human stem cell-derived models) hold great promise in generating mechanistic data for the thousands of untested chemicals (Crofton et al. 2012). This has been recently acknowledged by the EFSA Panel on Plant Protection Products Scientific Opinion on the developmental neurotoxicity potential for two pesticides, acetamiprid and imidacloprid (EFSA 2013). This panel articulated uncertainties in using in vitro studies, referring to the difficulties in setting health-based reference values, and stressing the fact that simple in vitro methods do not adequately model the complexity of neurodevelopmental processes. The panel acknowledged that in vitro assays may be regarded as complementary to animal testing, and as such, they could be incorporated into a DNT testing strategy to obtain mechanistic information or for purposes of screening/prioritization. The panel encouraged the development of criteria that would trigger submission of mandatory DNT studies, which could include development of an integrated and cost-effective, tiered testing strategy composed of robust, reliable and validated in vitro assays and alternative methods complementary to the in vivo test guideline 426 for assessing the developmental neurotoxicity potential of substances.

A major challenge in the use of all in vitro data, not just DNT data, is the uncertainty in extrapolation from in vitro concentrations to estimates of human exposure. Without estimates of human exposure, the risk assessment for many regulatory decisions is not possible. Judson et al. (2011) elaborated a framework for establishing biological pathway altering doses by using pharmaco-dynamic in vitro data together with pharmacokinetic modelling data. Wetmore et al. (2013) have developed a cost-effective model for estimating human daily doses from in vitro concentration, albeit with large confidence limits, and work is ongoing to reduce the uncertainty in these predictions as well as susceptible populations (Wetmore et al. 2014). This holds promise for the future that in vitro derived dose levels can be used as point of departure (POD) to calculate margins of exposure (MoE) (Thomas et al. 2013). MoE, defined as the ratio between the POD and estimated human exposure, provide an initial insight on the level of safety concern (Schilter et al. 2014) and will be invaluable in prioritizing chemicals for further investigation.

A promising approach to improved confidence in the use of in vitro DNT data by regulators is for species-to-species comparisons using IATA and ITS. OECD is currently developing a framework for the development and the use of IATAs, building on current activities on MOA and AOP. The IATA framework will provide guidance on how results from alternative approaches could be used and interpreted for characterizing (both qualitatively and quantitatively) the adverse effects in animals and humans and/or the environment, for the use in risk assessment and classification and labelling.

Based on current knowledge, available methods and assuming that hazard assessment must be balanced with management goals as well as time and resource constraints, in vitro DNT testing could significantly contribute to decision-making in the following ways:

- Reduce uncertainty in hazard estimates. In vitro DNT data can reduce uncertainties in hazard prediction by providing mechanistic data for specific chemicals, including extrapolation between rodents and humans. For example, cell-based assays analogous to key neurodevelopmental processes such as neural precursor commitment, neuronal and glial cell migration, proliferation, differentiation, synaptogenesis, neurite outgrowth, functional measurements of electrical activity, ratio between neuronal and glial cells provide a reliable mechanistic insight into a potential DNT’s effects (Bal-Price et al. 2012). Furthermore, these DNT specific measurements can be performed using neuronal/glial models derived from human pluripotent stem cells (embryonic or induced), circumventing the complex issues of species specificity (Fritsche 2014; Buzanska et al. 2009).
- To establish DNT as pivotal effect by determination of the sensitivity of developing neuronal cells in comparison with other cell types.
  Higher sensitivity/vulnerability of neurons than other cell types would either trigger the need for in vivo DNT data or justify the application of an additional uncertainty/safety factor on an existing health-based guidance value derived from a traditional animal database not covering neurodevelopmental studies. The application of a factor to address uncertainty regarding DNT potential would bring confidence regarding the suitability of the health-based guidance value to protect infant health.
- To optimize in silico and in vitro approaches for hazard characterization. It is increasingly acknowledged that the toxicological properties of in silico and in vitro tested chemical can be predicted based on their structural features, especially those with well-studied analogues, for example, through read across (Schilter et al. 2014; Patlewicz et al. 2014). The identification of analogues is not straightforward and should focus not only on chemical structure, but also on mechanism of action and/or biological activity/property (Schilter et al. 2014; Wu et al. 2010). Indeed, for a group of chemical analogues with members being documented as neurodevelopmental toxicants, in vitro investigations might improve the selection of the most relevant analogue(s) to be applied in read across. This requires demonstrating that the relevant specific neurodevelopmental effect is modelled adequately in vitro. In addition, knowledge built during the development of the AOPs specific for DNT will facilitate the construction of chemical categories for read across according to MIEs and KEs. The chemical classification can be further linked to a structure activity relationship to develop QSAR models specific for DNT effects.
- To trigger and tailor in vivo DNT studies. In the shorter term, in vitro data, together with signals from mandatory repeat-dose studies, could improve the design of the in vivo studies when they would be needed for regulatory purposes.

## Challenges in use of in vitro DNT in an AOP format

The AOP framework concept has brought novel opportunities for the application of alternative methods in chemical hazard identification. The challenge of utilizing such alternative approaches within the AOP concept lies first of all in the development of AOPs for DNT. As mentioned above (“Introduction of the AOP concept” section), an initial effort towards creation of possible DNT AOPs from the currently available literature has been initiated (Bal-Price et al. 2015), but there is still a large body of work to be done for accomplishing this task. A current challenge for DNT testing is the need to develop in vitro tests for MIEs that reliably predict AOs. DNT AOPs provide the framework that links MIEs to DNT AOPs via a series of KEs. Currently, alternative methods are evaluated individually by their predictivity with regard to their correct classification of positive and negative test compounds. When positioned within the AOP context, the usefulness of each assay is determined relatively to its place in the series of key events within an AOP. This approach offers the opportunity to verify that the in vitro method is part of a signalling pathway related to measurable KEs. For example, a pathway present, active and responsible for a KE in vivo should also be similarly responsive in an in vitro assay reflecting the same KE. Thus, the approach of placing in vitro methods into individual AOP contexts will increase the reliability of results from such well-characterized alternative assays because data are obtained on a sound knowledge base with clear determination of individual assay’s biological application domains. The criteria for establishing correlative and causative linkages in AOPs can be especially challenging in biological systems with high levels of biological complexity, such is the DNT. The developing brain holds spatiotemporal peculiarities as it contains many different interacting cell types within a variety of cellular structures and targets (Lieberman et al. 2008), many of which change their function over developmental time (Ben-Ari 2002). For example, neural tube closure or neural progenitor cell proliferation are affected at earlier stages than brain cortex maturation (Rice and Barone 2000). Thus, different MIEs may lead to the same adverse outcome, and several adverse outcomes may be caused by the same MIE, depending on exposure timing and developmental stage. In addition, there is species specificity in metabolism (Graham and Lake 2008) and toxicodynamics (Gassmann et al. 2010b; Heuer 2013) complicating animal–human extrapolation.

**Fig. 5:**
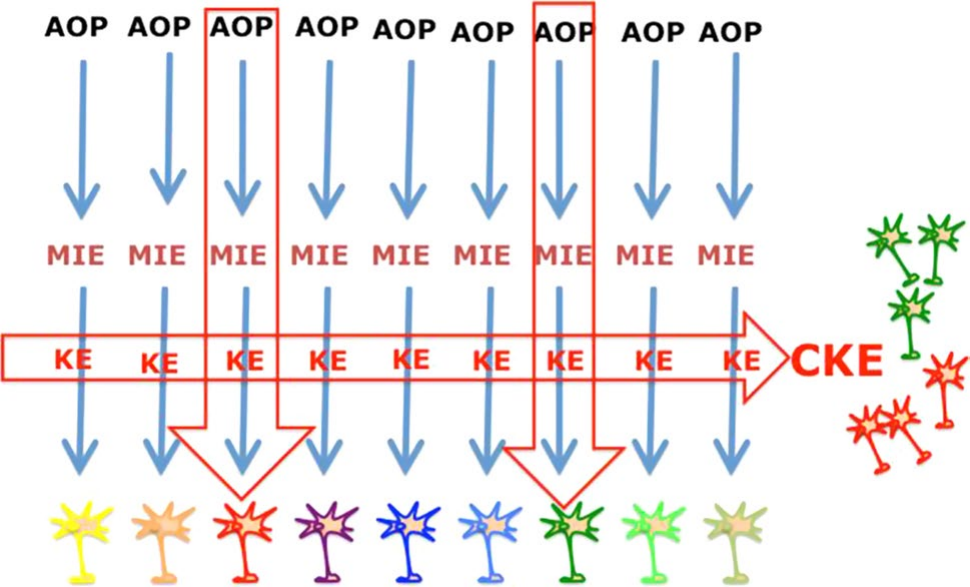
Concept of common key events (CKEs). CKEs are identical to KEs altered within multiple AOPs. When those are chosen as testing endpoints, the number of assays/AOPs can be drastically reduced

This complex situation may be simplified by focussing on common key events (CKEs, Fig. 5). An example of CKEs in DNT involve alterations in developmental processes critical for normal brain architecture development, such as neural crest cell proliferation and migration, neural stem/progenitor cell proliferation, migration and differentiation, neurite outgrowth, myelination, synapse and network formation and cell–matrix interactions. Compounds acting on different MIEs which alter one or more of these common developmental processes may be especially suited as common transversal read-outs for an array of different AOPs. For these endpoints, a variety of in vitro alternative assays are currently established and available (Bal-Price et al. 2012; Baumann et al. 2014; Fritsche 2014; Theunissen et al. 2012b). The challenge is for practical use of these CKEs in the AOP context. While they are clearly correlatively predictive for AOs, in that decreased number of neurons, mismigration of cell, or failure of synaptogenesis is all indicative of altered neurodevelopment; however, the causative links are currently lacking. This is not a failure of neurotoxicology research, but instead indicative of the lack of known pathobiology of most developmental neurological disorders. Therefore, it is crucial to understand that the state of the science currently provide the use for CKEs in correlative-based AOPs that allow only qualitative predictions of DNT AOs. Future research, including advances in developmental neurobiology, is needed, leading from causatively like developmental neurological signalling pathways to functional evaluation of brain development. The AOP framework empowers development of the CKE concept towards this goal.

## A path forward for developmental neurotoxicity testing

Any vision for the future of a scientific endeavour requires a clear articulation of the problem(s) for which solutions are needed. Problem formulation in research can be both difficult and time-consuming. For DNT testing, many of the dominant and pressing problems have been well articulated (Bal-Price et al. 2012; Crofton et al. 2012; Leist et al. 2013b; Smirnova et al. 2014). These problems include the need to (1) screen thousands of chemicals for bioactivity **(**Fig. 1; Fund 1997; NRC 1984) and (2) develop and use in vitro and in silico models based on human biology (EU 2013b; Leist et al. 2013a; NRC 2007).

Over the past decade, significant advances in the field of in vitro testing methods for DNT have led to the development and characterization of a variety of cellular-based systems (Baumann et al. 2014; Harrill et al. 2010; Hoelting et al. 2013; Hogberg et al. 2013; Honegger et al. 2011; Zimmer et al. 2012) and establishment of methods employing alternative model organisms (Avila et al. 2012; Cowden et al. 2012; Selderslaghs et al. 2013; Truong et al. 2014). New cellular models for assessing an array of DNT endpoints include primary neuronal cells from rodents, human and rodent tumour-derived neuronal cell lines, and stem-/progenitor cell-based neural cells from humans and rodents (Bal-Price et al. 2012; Crofton et al. 2012; Fritsche 2014). New alternative model species such as *C. elgans* and zebrafish have also been developed to study the impact of chemicals on the developing nervous system (Bal-Price et al. 2012; Crofton et al. 2012; de Esch et al. 2012; Padilla et al. 2011; Selderslaghs et al. 2013). More recently, the growing awareness that cells in a 3D context maintain physiological signalling much better than cells growing in a 2D format (Yamada and Cukierman 2007) led to the establishment of 3D models that recapitulate more physiological neurodevelopmental functions (e.g. Fritsche 2014; Pamies et al. 2014; Alépée et al. 2014; Baumann et al. 2014; Hoelting et al. 2013; Hogberg et al. 2013; Honegger et al. 2011; Moors et al. 2009). Recent publications also highlight new methods that allow for higher throughput testing of neuronal function in vitro using microelectrode arrays or “brain on a chip” technologies (Charkhkar et al. 2014; Kapucu et al. 2012; Novellino et al. 2011; Robinette et al. 2011; Ylä-Outinen et al. 2010). Together these methods demonstrate significant progress in methods development for DNT testing. However, a number of issues remain concerning application and predictability of such in vitro methods that must be addressed (Table 2).

**Table 2:** Summary of actions needed to build AOP-based in vitro DNT screening tools for regulatory use

Creation of putative AOPs for DNT by taking existing data on basic molecular developmental neuroscience as well as DNT into account that will foster:
Targeted generation of missing molecular-, cellular-, tissue- and organism-level data using in vitro and in vivo methods to develop validated AOPs
Identification of MIEs and/or KEs in priority AOPs for which cell models/alternative organisms must be generated
Generation of chemical training and testing sets for the use in assay development and validation
Generation of data sets for large numbers of chemical that allows qualification/validation of assay use that is “fit for purpose”, including:
Comparison of results across assays with similar endpoints
Comparison of results of different assays across chemicals
Development of in silico models (e.g. QSAR, docking models)
Development of a DNT alternative methods testing battery for the use in routine screening of new and existing chemicals
Development of predictive computational models based on AOPs that assess reliability of both individual test methods and the DNT testing battery, including:
Definition of model- and endpoint-specific quantitative cut-off values for delineating adversity
Development and incorporation of qualitative and quantitative species-specific differences in signalling pathway-driven guidance of developmental processes
Generation of case studies for use of AOP-based DNT screening data in regulatory decisions, including:
Use in multiple types of regulatory decision such as read across, prioritization for further testing and replacement of in vivo testing requirements

First and foremost of the remaining issues is the need to implement these test methods in order to develop data that will allow an assessment of their utility in screening for DNT. The state of the science for other areas of toxicology, such as developmental, reproductive and endocrine toxicity as well as carcinogenesis, has made immense progress. This includes the development of in vitro testing databases for hundreds of assays and thousands of chemicals in the ToxCast and Tox21 programs (Attene-Ramos et al. 2013; Kavlock et al. 2012). This has fostered development of computational models predicative of in vivo adverse outcomes (Martin et al. 2011; Rotroff et al. 2013; Sipes et al. 2011). The lack of adequate data sets for large numbers of chemicals from in vitro DNT assays has severely hampered the development of computational models. To date, a limited number of in vitro assays have used small sets of chemicals (20–75 chemicals) (Harrill et al. 2011; Krug et al. 2013; Novellino et al. 2011; Zimmer et al. 2014).

These and other available assays need to be used to begin screening larger chemical libraries (e.g. Tox21/ToxCast). However, moving from methods development to use in screening large sets of chemicals for DNT requires caution (Kadereit et al. 2012). Performance and predictive value of an assay requires determination of whether the assay is “fit for purpose” and different sets of chemicals may be needed depending on the use of the data (Crofton et al. 2011; Judson et al. 2013; Kadereit et al. 2012). Chemical libraries should also contain chemicals known or suspected of causing DNT effects. Lists of such chemicals have been proposed (Grandjean and Landrigan 2014; Kadereit et al. 2012).

As mentioned above, another major issue in the development and use of in vitro data for regulatory purposes is the lack of well-described and codified AOPs for developmental neurotoxic outcomes. To date, only a limited number of peer-reviewed AOPs are available for DNT and these are limited to endocrine-related MIEs (Crofton and Zoeller 2005; Zoeller and Crofton 2005).

A recent review (Bal-Price et al. 2015) has highlighted the need for the development of more AOPs specific for neurotoxic and developmental neurotoxic outcomes, and provides a number of putative AOPs for future development. This lack of AOPs hampers the ability to correlatively or causatively link data from in vitro assays that target MIEs of upstream key events to adverse outcomes (Ankley et al. 2010). These kinds of linkages are necessary to build confidence for the use in regulatory decisions. Creation of AOPs for DNT must take into account the temporal and dose dynamics critical for neurodevelopment processes. This is needed to ensure that in vitro and alternative species models encompass the critical processes necessary for brain development (Coecke et al. 2007). This type of approach will also assist in the targeted generation of missing molecular-, cellular-, tissue- and organism-level data using in vitro and in vivo methods to develop AOPs. AOP development will also foster identification of MIEs and/or key events in AOPs for which in vitro assays need to be developed.

As discussed earlier, normal development of the nervous system is a complex set of interactions between different neuronal and glial cell types and developmental processes that are temporally and spatially regulated (neuroepithelial differentiation from embryonic stem cells, neural crest cell migration, neural stem/progenitor cell proliferation, radial glia migration, differentiation into neurons and glial cells, neuronal migration, neurite outgrowth, dendrite development, synaptogenesis and network formation). Therefore, assays, especially those employing a single cell type cultures, are unlikely to be able to correctly identify DNT potential for all chemicals. A number of approaches are currently being pursued including development of a battery of assays using multiple neuronal and glial cell types (Coecke et al. 2007) as well as using three-dimensional multi-cell cultures with read-outs for multiple neurodevelopmental processes (Alépée et al. 2014; Baumann et al. 2014; Zurich et al. 2002; Monnet-Tschudi et al. 2000). Recently, new microfluidic organs-on-chips methods have been developed that more accurately simulate tissue- and organ-level physiology (Bhatia 2014). These new approaches will not only provide great insight into the complex dynamics of neurodevelopment, but also allow more realistic testing scenarios. More work is urgently needed to produce testing data that will allow comparisons of the predictive capacity across assays and batteries of assays with the goal of developing a set of tests assembled into testing batteries that cover pathways and processes of brain development as comprehensively as possible. Development and use of batteries of in vitro assays for testing chemicals will be a huge step forward into DNT evaluation.

Another major need for the development and the use of DNT in vitro methods is the generation of case studies for use of AOP-based DNT screening data in regulatory decisions. While at the present time there may not be enough data available, planning should begin that includes discussions between assay developers, assay users and regulatory authorities. It is critical to engage regulators to ensure a fit-for-purpose approach while these methods are being developed. For example, regulatory authority may accept a higher level of scientific “uncertainty”, in both the methods and the resulting data, when making read across or prioritization decisions (Judson et al. 2013). Replacement of in vivo tests with in vitro alternatives will require that methods undergo a higher level of validation and result in data with much less uncertainty. The purpose of the current workshop was to begin these discussions, generate ideas and interactions that will foster and speed development, use and acceptance of alternative methods for DNT testing.
